# Proteomic differences among patients with heart failure taking furosemide or torsemide

**DOI:** 10.1002/clc.23733

**Published:** 2022-01-11

**Authors:** Lauren B. Cooper, Scott Bruce, Mitchell Psotka, Robert Mentz, Rachel Bell, Stephen L. Seliger, Christopher O'Connor, Christopher deFilippi

**Affiliations:** ^1^ Department of Cardiology Donald and Barbara Zucker School of Medicine at Hofstra/Northwell Manhasset New York USA; ^2^ Inova Heart & Vascular Institute Inova Fairfax Hospital Falls Church Virginia USA; ^3^ Department of Statistics, Volgenau School of Engineering George Mason University Fairfax Virginia USA; ^4^ Department of Medicine Duke University School of Medicine Durham North Carolina USA; ^5^ Department of Medicine University of Maryland School of Medicine Baltimore Maryland USA

**Keywords:** biomarkers, heart failure, proteomics

## Abstract

**Background:**

Loop diuretics are commonly used for patients with heart failure (HF) but it remains unknown if one loop diuretic is clinically superior.

**Hypothesis:**

Biomarkers and proteomics provide insight to how different loop diuretics may differentially affect outcomes.

**Methods:**

Blood and urine were collected from outpatients with HF who were taking torsemide or furosemide for >30 days. Differences were assessed in cardiac, renal, and inflammatory biomarkers and soluble protein panels using the Olink Cardiovascular III and inflammation panels.

**Results:**

Of 78 subjects, 55 (71%) were treated with furosemide and 23 (29%) with torsemide, and 25 provided a urine sample (15 treated with furosemide, 10 with torsemide). Patients taking torsemide were older (68 vs 64 years) with a lower mean eGFR (46 vs 54 ml/min/1.73 m^2^), a higher proportion were women (39% vs 24%) and Black (43% vs 27%). In plasma, levels of hs‐cTnT, NT‐proBNP, and hsCRP were not significantly different between groups. In urine, there were significant differences in urinary albumin, β‐2M, and NGAL, with higher levels in the torsemide‐treated patients. Of 184 proteins testing in Olink panels, in plasma, 156 (85%) were higher in patients taking torsemide but none were significantly different after correcting for false discovery.

**Conclusions:**

We show differences in urinary biomarkers but few differences in plasma biomarkers among HF patients on different loop diuretics. Olink technology can detect differences in plasma protein levels from multiple biologic domains. These findings raise the importance of defining differences in mechanisms of action of each diuretic in an appropriately powered study.

## 1 INTRODUCTION

The prevalence of heart failure (HF) is increasing in the United States and worldwide. Loop diuretics including furosemide and torsemide remain a foundation of therapy for patients with HF and fluid retention. While furosemide is much more commonly used, it remains unknown if one loop diuretic is clinically superior for patients with HF.^1^, ^2^, ^3^ In these patients with HF, renal and cardiac function are closely linked. Renal function is monitored during treatment as many therapies have the potential to worsen renal function, though the mechanisms and long‐term impact of this worsening renal function (WRF) are uncertain. The manner by which torsemide and furosemide may differentially alter the trajectory of renal function and ultimately affect outcomes for patients with HF remains undetermined.^4^, ^5^ Soluble protein biomarkers are surrogates of physiological status and risk assessment and are used as clinical cornerstones for the diagnosis and prognosis of patients with HF. However, traditional targeted biomarker measurements of one to several soluble proteins representing a limited number of mechanisms or end end‐organ sequela may not be able to identify the mechanistic differences that could provide insight into potential outcome related differences between the diuretics. Until recently, technological limitations and cost restricted the measurement of broad panels of low‐abundance proteins in large cohorts and clinical trial studies with the analytical precision to elucidate subtle biologic differences. Targeted discovery proteomics, inclusive of high‐fidelity assays providing tightly reproducible measures of multiple soluble proteins, provides an opportunity to follow subtle longitudinal changes by measuring low abundance soluble proteins that track with the natural progression of disease or reflect the influence of an intervention. Antibody‐oligonucleotide technology by Olink is a novel method of targeted discovery proteomics that is gaining acceptance as an accurate and affordable methodology for high throughput proteomics in large populations.^6^ Whether this technology can help inform the mechanism by which torsemide and furosemide affect renal function and outcomes in patients with heart failure remains unknown. In this pilot study, we sought to identify and characterize WRF events during the recovery from acute HF using a panel of urinary biomarkers of tubular injury and examine whether diuretic strategy influences the change in these renal tubular injury biomarkers. Additionally, we sought to compare traditional biomarkers in patients with HF on long‐term diuretic therapy and determine the utility of this Olink ELISA‐based technology in assessing differences between diuretics to inform future trial design.

## 2 METHODS

Patients with a clinical diagnosis of HF and who were taking either torsemide or furosemide for >30 days, and were willing to provide a blood and urine sample, were enrolled from a single integrated health network. Participants included patients prospectively recruited between September 2019 and January 2020 at 1 of 3 clinical sites or those who had biospecimens previously frozen from an institutional biobank from May 2015 through October 2016. Patients were excluded if they were on either renal dialysis, had undergone renal transplantation, had a left ventricular assist device, or had undergone cardiac transplantation.

In plasma, we measured a panel of established single analytically robust commercial biomarkers of cardiac injury, strain, renal function, and generalized inflammation and fibrosis with known prognostic roles in patients with HF (high sensitive cardiac troponin T [hs‐cTnT], amino terminal pro B‐type natriuretic peptide [NT‐proBNP], growth differentiation factor 15 [GDF‐15], Cystatin C, high sensitive C‐reactive protein [hsCRP], and interleukin 6 [IL6]).^7^, ^8^, ^9^, ^10^, ^11^, ^12^, ^13^, ^14^, ^15^, ^16^, ^17^, ^18^, ^19^ Biomarkers were analyzed on either the Roche Cobas e602 (hs‐cTnT, NT‐proBNP, GDF‐15) or Siemens Dimension EXL (Cystatin C, hsCRP, and creatinine).

In urine, urinary concentrations of the following biomarkers were compared between groups: albumin, β‐2 microglobulin (β2M), Cystatin C, epidermal growth factor (EGF), neutrophil gelatinase‐associated lipocalin (NGAL), osteopontin (OPN), and uromodulin (UMOD). The selection of biomarkers was designed to mimic those selected from the SPRINT kidney tubular health ancillary project and are described in detail elsewhere, and are known to be markers of renal injury and fibrosis.^1^ Biomarkers were analyzed using the Meso Scale Discovery Kidney Injury V (human) kit and concentrations were compared using a nonparametric Wilcoxon test.

Targeted discovery proteomics were analyzed by Olink (Watertown, MA). Using two panels (Cardiovascular III [CVD III] and Inflammation) in the domains of cardiovascular disease and generalized inflammation, we tested for differences in 184 soluble proteins. The Olink antibody‐oligonucleotide technology has overcome analytical precision issues of prior generation multiplex ELISA panels.^20^, ^21^ Proximity Extension Assay (PEA) panels have been created to represent loosely conjoined proteins related to the physiology or organ of interest. Through the use of oligonucleotide‐labeled antibody probe pairs which bind to each protein, PEA technology permits the simultaneous assessment of multiple proteins maintaining the precision of a duel epitope ELISA without the loss of specificity of earlier generation multiplex assay systems. The PEA assay reports levels as NPX units representing protein concentration measured on the log 2 scale (i.e., an increase in NPX of 1 unit corresponds to a doubling of protein concentration). The proteins can be curated by known biologic roles, which can include more than 1 domain and they are shown in Supplemental Figure 1.

Baseline participant characteristics are presented as mean (SD) for continuous variables and as number (percent) for categorical variables for all participants and separately for participants in each treatment group. Comparisons were made using 2‐sample *t*‐tests for continuous variables and χ2 test for categorical variables. Baseline differences in mean protein levels, measured on the log 2 scale and divided into the 2 panels described above, between participants receiving torsemide versus furosemide were examined using 2‐sample *t*‐tests. For traditional blood and urine biomarkers, concentration levels were measured on the linear scale and summarized by their medians and first and third quartiles. Given the non‐normal distributions of biomarker concentration levels, the non‐parametric Wilcoxon rank test was used to compare levels between the two diuretic groups.

T‐statistics for treatment differences between groups for each protein were calculated. A nominal *p*‐value of <0.05 was considered significant. False discovery rate (FDR) adjusted *p*‐values were calculated accounting for the number of proteins examined and grouping into panels. We controlled for false discovery using the method of Benjamini–Hochberg.^22^ FDR *p*‐values of less than 0.05 were considered statistically significant.

The Inova Health System institutional review board approved this study. Prospectively enrolled patients signed an informed consent. Biobank samples were previously approved for exploratory analysis.

## 3 RESULTS

We analyzed plasma from 78 patients, 52 patients who were prospectively consented and enrolled at 1 of 3 clinic sites and 26 samples from an institution‐specific biobank. Of those patients, 25 provided a urine sample (15 treated with furosemide, 10 with torsemide). In this nonrandomized cohort, 55 subjects were treated with furosemide and 23 with torsemide (Table 1). The mean age was 66 years, with 28% female and 32% black subjects. The torsemide group was older, included a higher proportion of women and Black patients compared to furosemide. Patients taking torsemide also had a lower estimated glomerular filtration rate, and lower systolic blood pressure.

**Table 1:** Biomarker pilot study patient characteristics

Variable	All (*N* = 78)	Furosemide (*N* = 55)	Torsemide (*N* = 23)
Age (years), mean (SD)	64.8 (13.7)	63.7 (14.4)	67.5 (11.8)
Sex			
Men	56 (72%)	42 (76%)	14 (61%)
Women	22 (28%)	13 (24%)	9 (39%)
Race			
White	44 (56%)	34 (62%)	10 (43%)
Black	25 (32%)	15 (27%)	10 (43%)
Other/unknown	9 (12%)	6 (11%)	3 (13%)
EF (%,) mean (SD)	34.7 (18.1)	33.8 (17.3)	36.7 (20.1)
eGFR (ml/min/1.73 m2), mean (SD)	51.5 (14.8)	53.9 (14.4)	45.7 (14.7)
Systolic BP (mmHg), mean (SD)	116.9 (21.1)	117.5 (21.4)	115.4 (20.5)
HR (bpm), mean (SD)	77.2 (15.9)	76.5 (17.5)	78.9 (11.6)
Weight (kg), mean (SD)	91.7 (22.6)	92.2 (23.5)	90.4 (20.6)
NYHA			
Missing	17 (22%)	13 (24%)	4 (17%)
1 or 1–2	13 (17%)	8 (15%)	5 (22%)
2 or 2C or 2–3	20 (26%)	13 (24%)	7 (30%)
3 or 3B or 3–4	20 (26%)	17 (31%)	3 (13%)
4	8 (10%)	4 (7%)	4 (17%)

Demographics for the urine cohort were similar with an average age of 66 ± 14 years old, 80% male, and 72% white. The mean estimated glomerular filtration rate was lower in the torsemide group compared to the furosemide group (39.1 ± 11.8 vs 51.4 ± 12.0 ml/min/1.73m^2^), and mean weight was higher (91.4 vs 88.7 kg). One patient in the furosemide group had marked outliers in several biomarkers and was excluded from the urinary analysis.

In blood, NT‐proBNP levels were similar between the torsemide and furosemide group (median 1298 vs 1259 pg/ml, p = 0.84; Table 2). In addition, there was no difference between patients on torsemide and furosemide for biomarkers hsCRP, IL6, hs‐cTnT, or GDF15. There were higher levels of Cystatin C for those on torsemide compared with furosemide (1.62 vs 1.19 mg/L, *p* = 0.007) consistent with the lower creatinine based eGFR in this group.

**Table 2:** Biomarker concentrations

Biomarker	Torsemide *N* = 22	Furosemide *N* = 56	*p*‐value
	Median (25th, 75th)	Median (25th, 75th)	
hsCRP (mg/L)	4.25 (2.39, 9.30)	3.12 (0.83, 7.43)	.20
Cystatin C (mg/L)	1.62 (1.32, 1.92)	1.19 (1.00, 1.58)	.007
IL6 (pg/ml)	4.79 (2.81, 8.19)	4.93 (3.05, 9.05)	.81
NTproBNP (pg/ml)	1298 (437, 3532)	1259 (480, 2942)	.84
hs‐cTnT (ng/L)	28 (18, 51)	20 (14, 42)	.23
GDF15 (pg/ml)	3158 (2048, 6025)	2399 (1365, 4089)	.31

In urine, the concentrations of urinary biomarkers among torsemide versus furosemide treated patients are shown in Supplemental Figure 2. Among the seven biomarkers, significant between‐group differences were found in the distribution of urinary albumin (*p* = 0.001), β‐2M (*p* = 0.03), and NGAL (*p* = 0.04), with higher levels in the torsemide‐treated patients.

**Figure 1:**
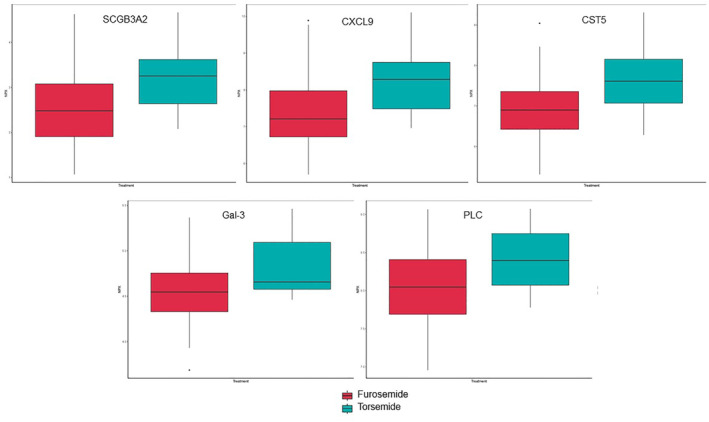
Select box and whisker plots for differences in proteins from patients on furosemide versus torsemide

**Figure 2:**
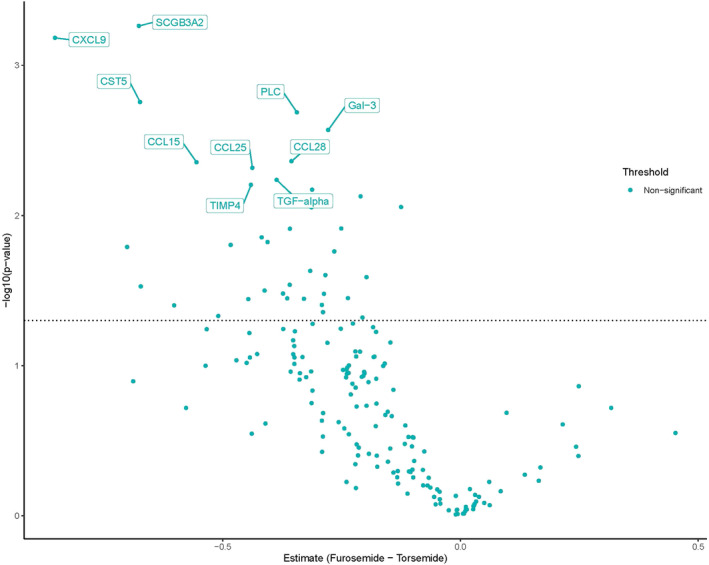
Furosemide versus torsemide in plasma. X‐axis represents difference in protein levels expressed as NPX units (comparable to the natural log of the concentrations). Values left of zero indicate the protein level was higher in torsemide patients. The Y‐axis is the log of the statistical significance with the dashed horizontal line indicating the level of unadjusted significance (i.e., *p* = 0.05)

For targeted discovery proteomics for the plasma samples, in the two panels, there were 40 proteins that by *t*‐test, uncorrected for false discovery, were different between patients taking furosemide versus torsemide (Table 3). Despite 40 proteins meeting the threshold for nominal significance, none met significance after correction for false discovery. Box plots for the five proteins with the largest difference are shown in Figure 1. All measured proteins from plasma are shown in a volcano plot in Figure 2. A smaller proportion of the proteins were detected in urine and at lower concentrations than plasma.

**Table 3:** Differences in proteins between torsemide and furosemide

Assay	Panel	estimate	Furosemide	Torsemide	Unadjusted *p* value
SCGB3A2	CVDIII	−0.68	2.54	3.22	<.001
CXCL9	INF	−0.85	7.40	8.25	<.001
CST5	INF	−0.67	6.94	7.61	.002
PLC	CVDIII	−0.34	8.06	8.41	.002
Gal‐3	CVDIII	−0.28	4.55	4.82	.003
CCL28	INF	−0.35	2.07	2.42	.004
CCL15	CVDIII	−0.55	7.67	8.23	.004
CCL25	INF	−0.44	6.35	6.78	.005
TGF‐alpha	INF	−0.39	3.04	3.42	.006
TIMP4	CVDIII	−0.44	4.64	5.08	.006
FGF‐5	INF	−0.31	0.98	1.29	.007
CSF‐1	INF	−0.21	9.91	10.12	.007
TNF	INF	−0.31	3.11	3.42	.009
VEGFA	INF	−0.37	11.01	11.38	.009
IL33	INF	−0.12	0.28	0.40	.009
IL‐15RA	INF	−0.31	1.22	1.53	.009
SPON1	CVDIII	−0.25	1.15	1.40	.01
MCP‐3	INF	−0.36	1.73	2.09	.01
U‐PAR	CVDIII	−0.42	5.22	5.64	.01
CCL23	INF	−0.41	10.04	10.44	.015
TFF3	CVDIII	−0.48	6.70	7.19	.02
FABP4	CVDIII	−0.70	5.58	6.28	.02
CXCL16	CVDIII	−0.26	5.67	5.94	.02
FAS	CVDIII	−0.32	5.71	6.02	.02
Notch 3	CVDIII	−0.28	5.27	5.56	.025
LIF‐R	INF	−0.20	3.94	4.14	.03
CX3CL1	INF	−0.36	4.10	4.46	.03
PI3	CVDIII	−0.67	3.06	3.74	.03
IL8	INF	−0.41	5.39	5.80	.03
LTBR	CVDIII	−0.37	3.84	4.21	.03
TNFRSF10C	CVDIII	−0.29	4.50	4.79	.03
KLK6	CVDIII	−0.24	4.08	4.32	.04
CDCP1	INF	−0.36	2.94	3.30	.04
SLAMF1	INF	−0.33	2.39	2.72	.04
OPN	CVDIII	−0.45	8.29	8.74	.04
MCP‐2	INF	−0.29	8.47	8.76	.04
IL‐17C	INF	−0.60	3.08	3.68	.04
ARTN	INF	−0.29	0.47	0.76	.04
IGFBP‐2	CVDIII	−0.51	8.62	9.13	.047
TFPI	CVDIII	−0.21	8.92	9.13	.048

## 4 DISCUSSION

In this single center, prospective pilot study of ambulatory patients with HF receiving torsemide or furosemide, we analyzed both blood and urine biomarkers to assess for differences in patients receiving these diuretics and tested the feasibility of utilizing Olink targeted discovery proteomics to detect differences. We demonstrated that there are marked differences in three urinary biomarkers between patients, but only 1 significant differences in many commonly tested blood biomarkers, highlighting the need for methods that can search more broadly and detect more subtle, but potentially meaningful differences between patients on these drugs. In exploring the Olink methodology of targeted discovery proteomics, we showed that this technology can potentially detect subtle differences between groups in plasma but with a low yield of protein detection in urine.

While there is substantial supportive evidence for many guideline‐directed therapies in HF, there are limited data regarding one of the most commonly used classes of drugs in HF, loop diuretics.^23^ Despite suggestive preclinical mechanistic and clinical data, robust randomized controlled trial data are only now being generated.^24^, ^25^, ^26^, ^27^, ^28^, ^29^

Loop diuretics have been shown to activate the renin‐angiotensin‐aldosterone system (RAAS), leading to deleterious effects and progression of HF.^30^ However, while furosemide increases circulating aldosterone levels, torsemide has anti‐aldosteronergic and anti‐kaliuretic effects, with reductions in aldosterone secretion and receptor binding, leading to improvements in ventricular wall stress, decreased sympathetic nervous system activity, direct vasodilation, and multifactorial reductions in detrimental myocardial fibrosis.^31^, ^32^, ^33^, ^34^, ^35^, ^36^, ^37^, ^38^, ^39^, ^40^, ^41^ Additionally, torsemide has better bioavailability and potency than furosemide which may contribute to superior efficacy, and outcomes including improved functional status, quality of life, and reduced hospitalizations.^42^, ^43^, ^44^ The TORIC study, the largest study to date comparing torsemide and furosemide, was an open‐label post‐marketing surveillance study; although it showed more functional improvement and less mortality in patients receiving torsemide, these results must be taken in the context of the non‐randomized study design.^45^ Despite suggestions from open‐label trials that patients treated with torsemide have better outcomes, results from other studies demonstrated no benefit.^27^, ^28^ Two recent meta‐analyses showed that torsemide was associated with improved functional status compared to furosemide, but clinical outcomes were similar between the diuretics.^25^, ^29^

Renal function likely plays a role in clinical outcomes for HF patients on diuretic therapy. In the acute HF setting, WRF has been associated with adverse outcomes independent of co‐morbidities and LVEF.^2^ However, recent studies have questioned the clinical implications and consequences of WRF in the acute HF setting, with no excess mortality observed in those with WRF unless accompanied by evidence of persistent vascular congestion.^3^

In the current study, significantly higher levels of urinary biomarkers albumin, β2 microglobulin, and NGAL in the torsemide group suggest that there may be a greater degree of renal tubular injury and/or dysfunction in patients who are treated with torsemide compared with furosemide, though the clinical significance of these differences in the chronic HF population is unknown.

Whether the choice of loop diuretic alters the trajectory of renal function or contributes to renal tubular injury during the treatment of acute or chronic HF is unknown. In principal, more rapid natriuresis and intra‐renal anti‐aldosteronergic effects associated with torsemide versus furosemide could lead to sub‐acute decline in renal function through hemodynamic effects; if diuretic‐induced intra‐renal hypoperfusion is persistent and severe, renal tubular injury may occur.^4^ Alternatively, in cases of persistent venous congestion, ongoing effective natriuresis could plausibly reduce the risk of WRF or even cause modest improvements in renal filtration function. The prognostic importance of urinary tubular injury biomarkers in chronic HF is uncertain.

Much of the translational data regarding the influence of torsemide on RAAS inhibition and aldosterone antagonism comes from animal studies and human translational data typically based on a highly targeted selection of soluble biomarkers with inconsistent findings.^41^ Translation of findings in animals to small human observational studies while measuring single or a small panel of proteins has restricted opportunities to understand the impact of demographic and clinical phenotypes. This has limited the generalizability of prior mechanistic findings. Highlighting the limited human translational insights for diuretic choice based on single targeted biomarkers, we found little difference in blood biomarker levels differentiating the two groups based on diuretic use, with the exception of higher levels of Cystatin C for those on torsemide consistent with the lower creatinine based eGFR in this group. These results emphasize the need for an expanded proteomic analysis to characterize differences in biologic mechanisms that could impact prognosis beyond what would be identified with established soluble biomarkers.

Until recently, technological limitations and cost restricted the measurement of broad panels of low‐abundance proteins in large cohorts and clinical trials with the analytical precision to follow small, but potentially important biologic longitudinal change. Until recently, more agnostic “discovery” or “targeted” based proteomics approaches to cardiovascular disease progression or response to treatment were limited respectively by analytical precision/cost or a limited portfolio of immunoassays that could be tested on blood samples from large cohort studies. Discovery proteomics by mass spectroscopy, which has been available for some time, can be used to discover thousands of proteins. However, mass spectroscopy has limited sensitivity to detect many of the cardiac and renal related mechanistic proteins of interest that exist in low abundance in plasma (i.e., concentrations of pg/ml) Moreover, a notable limitation for mass spectroscopy when using blood is that higher abundance proteins (i.e., albumin) typically need to be removed to detect lower abundance proteins, but many low‐abundance proteins bind to the higher abundance proteins and may be co‐removed in the process.^38^ Recently, two novel technologies have been validated in cardiovascular disease to measure hundreds to thousands of proteins.^39^, ^40^, ^41^, ^42^, ^43^ The single aptamer based detection approach has the potential to provide a broad coverage of targeted discovery proteins, but recent concerns have been raised with respect to the specificity associated with this technology.^44^ This current study evaluated the feasibility of the other novel technology, antibody‐oligonucleotide technology by Olink. We have shown the Olink technology is capable of detecting differences in plasma in protein concentrations from multiple biologic domains relevant to cardiac pathophysiology.

Our study had several limitations. Given the cross‐sectional design, modest sample size, and likelihood of confounding by indication, it is not possible to determine that torsemide results in higher levels of blood and urine soluble inflammatory and fibrosis proteins. The urine biomarker analysis was further limited by lack of available urinary concentration measurements. However, these data raise the importance of defining differences in biologic sequela based on mechanisms of action of each diuretic in an appropriately powered ancillary study within a randomized controlled trial.

In conclusion, this analysis shows that there are limited differences in plasma biomarkers but several significant difference in some urinary biomarkers among HF patients on chronic furosemide therapy versus torsemide therapy. Further, the Olink technology is capable of detecting differences in protein concentrations from multiple biologic domains relevant to cardiac pathophysiology in patients on torsemide compared with furosemide. Further study is needed to elucidate the underlying mechanisms for the differences between these groups.

## CONFLICT OF INTEREST

The authors declare no conflicts of interest.

## Supporting information


**Supplemental Figure 1** The classification of proteins according to biological process. Reproduced with permission

**Supplemental Figure 2** Urinary biomarkers are shown as the median, interquartile range (box) and 5%–95% range (lines)

## Data Availability

The data that support the findings of this study are available on request from the corresponding author. The data are not publicly available due to privacy or ethical restrictions.
