# Supplementary figures and images for: Proteomic differences among patients with heart failure taking furosemide or torsemide
Source: Clin Cardiol. 2022 Jan 11;45(3):265–72. doi: 10.1002/clc.23733 (PMC8922525; doi:10.1002/clc.23733)

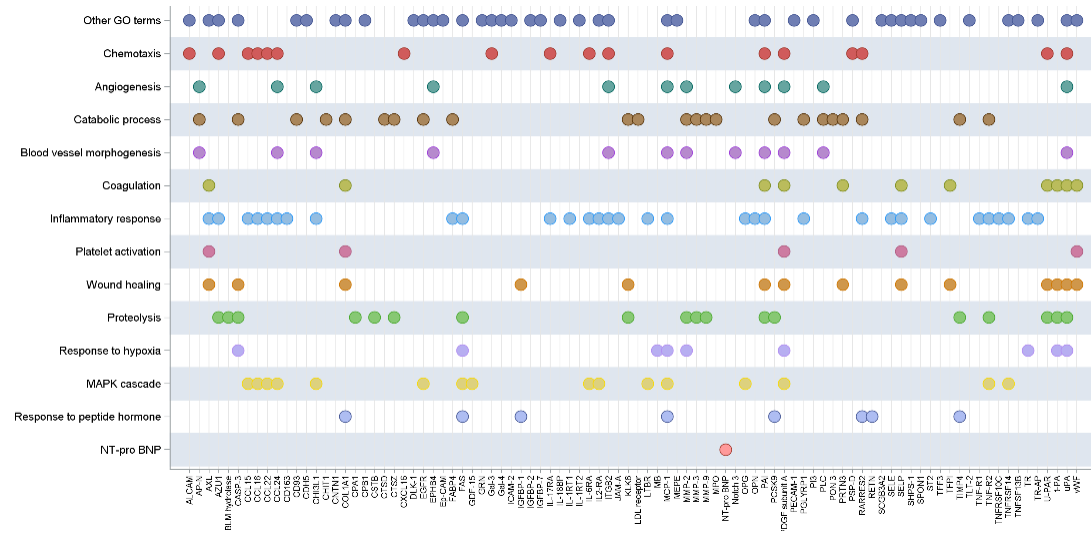

Supplement: Supplementary file 1 — Supplemental Figure 1 The classification of proteins according to biological process. Reproduced with permission [file CLC-45-265-s002.pdf]
