# Supplementary material for: Proteomic differences among patients with heart failure taking furosemide or torsemide
Source: Clin Cardiol. 2022 Jan 11;45(3):265–72. doi: 10.1002/clc.23733 (PMC8922525; doi:10.1002/clc.23733)
Supplement: Supplementary file 2 — Supplemental Figure 2 Urinary biomarkers are shown as the median, interquartile range (box) and 5%–95% range (lines) [file CLC-45-265-s001.pdf]

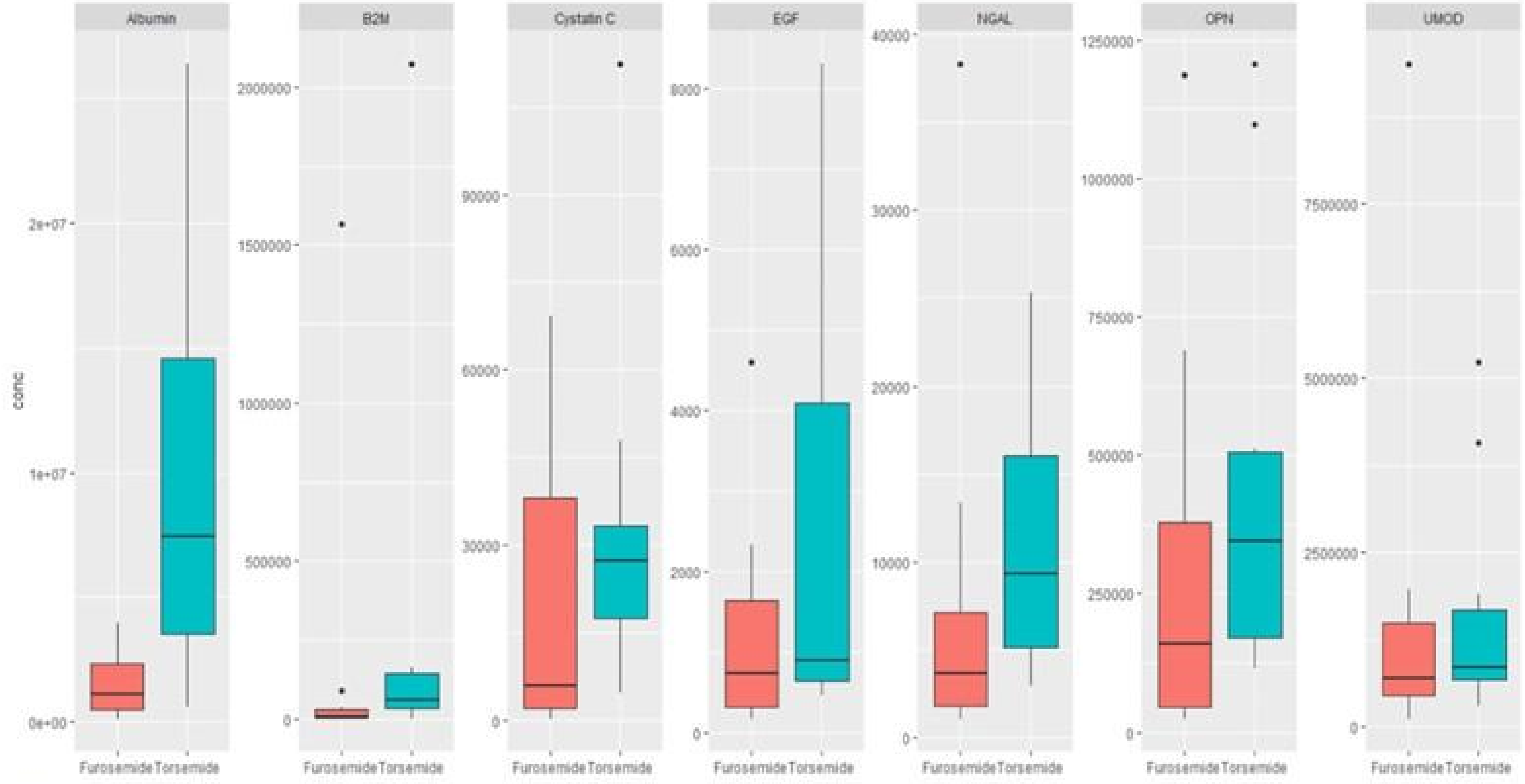

	Albumin		B2M		Cystatin C		EGF		NGAL		OPN		UMOD	
Mean concentration (pg/mL) in urine $x10^3$	1512.9	9368.3	136.97	269.21	19.364	33.129	1.1983	2.4686	6.700	11.429	280.06	458.54	1481.6	1584.1
p-value	0.001		0.03		0.15		0.21		0.04		0.15		0.58	

Figure. Urinary concentrations of renal tubular injury biomarkers after at least 30 days of diuretic therapy.
